# Citric Acid Enhanced Copper Removal by a Novel Multi-amines Decorated Resin

**DOI:** 10.1038/srep09944

**Published:** 2015-05-12

**Authors:** Chen Ling, Fuqiang Liu, Zhiguo Pei, Xiaopeng Zhang, Mengmeng Wei, Yanhong Zhang, Lirong Zheng, Jing Zhang, Aimin Li, Baoshan Xing

**Affiliations:** 1 State Key Laboratory of Pollution Control and Resource Reuse, School of the Environment, Nanjing University, Nanjing 210023, P. R. China; 2 State Key Laboratory of Environmental Chemistry and Ecotoxicology, Research Center for Eco-Environmental Sciences, Chinese Academy of Sciences, Beijing 100085, China; 3 Beijing Synchrotron Radiation Laboratory, Institute of High Energy Physics, Chinese Academy of Sciences, Beijing 100049, China; 4 Stockbridge School of Agriculture, University of Massachusetts, Amherst, Massachusetts 01003, United States

## Abstract

Cu removal by a novel multi-amines decorated resin (PAMD) from wastewater in the absence or presence of citric acid (CA) was examined. Adsorption capacity of Cu onto PAMD markedly increased by 186% to 5.07 mmol/g in the presence of CA, up to 7 times of that onto four commercial resins under the same conditions. Preloaded and kinetic studies demonstrated adsorption of [Cu-CA] complex instead of CA site-bridging and variations of adsorbate species were qualitatively illustrated. The interaction configuration was further studied with ESI-MS, FTIR, XPS and XANES characterizations. The large enhancement of Cu adsorption in Cu-CA bi-solutes systems was attributed to mechanism change from single-site to dual-sites interaction in which cationic or neutral Cu species (Cu^2+^ and CuHL^0^) coordinated with neutral amine sites and anionic complex species (CuL^−^ and Cu_2_L_2_^2−^) directly interacted with protonated amine sites via electrostatic attraction, and the ratio of the two interactions was approximately 0.5 for the equimolar bi-solutes system. Moreover, commonly coexisting ions in wastewaters had no obvious effect on the superior performance of PAMD. Also, Cu and CA could be recovered completely with HCl. Therefore, PAMD has a great potential to efficiently remove heavy metal ions from wastewaters in the presence of organic acids.

Heavy metal ions (HMIs) in wastewaters have always been major concerns, because they are toxic, persistent and bioaccumulative^1^,^2^,^3^. Wastewaters from industries such as electroplating, circuit board printing and mineral processing are the main sources of HMIs. These wastewaters also contain a great deal of organic acids (OAs) which are brought in during flotation, plating and rinsing^4^,^5^,^6^. Various soluble complex species can form over a wide range of pH, decreasing the efficiency of traditional removal methods such as chemical precipitation and coagulation^6^,^7^,^8^. Thus, innovative methods are needed for the increasingly stringent wastewater regulation^9^. Approaches with membrane and electrolysis often face high operation costs, while hybrid techniques involving the combination of advanced oxidation, coagulation and/or chemical precipitation have secondary pollution and long processes^10^,^11^,^12^. By contrast, adsorption, a relative simple, green and recyclable way, is considered as a promising technology for removing HMIs from wastewaters containing OAs^13^,^14^.

In the past years, many kinds of adsorbents were developed but suffered the following drawbacks: (1) OAs markedly suppressed HMIs adsorption due to either competing for the same adsorption sites or forming complex species with lower adsorption affinity^9^,^15^; (2) HMIs was hardly adsorbed from wastewater without the presence of high load of OAs^16^; and (3) application of soil- and bio-materials was much restricted because of their low capacity, poor mechanical strength or unsatisfactory recyclability^15^,^17^,^18^,^19^. Therefore, it is of significance to develop a stable adsorbent widely suitable for high removal and recovery of HMIs from wastewaters even in the presence of OAs.

Recently, several adsorbents with abundant amine groups showed good capacity for HMIs in the presence of OAs. Lu *et al*.^20^ and Guzman *et al*.^21^ reported chitosan had a larger capacity for Cu (maximum ~3.5 mmol/g) from wastewaters containing citrate or tartrate. Makton *et al*.^22^ found the presence of citric acid enhanced Cu adsorption of polyethyleneimine (PEI) modified agarose from sodium acetate buffered solutions. However, to date, adsorption mechanisms of these amine adsorbents for HMIs from HMIs-OAs bi-solutes systems in contrast with single solute systems are not well understood, though, such information is crucial to guide designing adsorbents and wastewater treatment process. For instance, Guzman *et al*.^21^ concluded that Cu adsorption was driven by the electronic attraction of anionic Cu complexes and protonated amine groups, and suppressed by the competition of anionic Cu-free ligands. Differently, Markton *et al*.^22^ proposed two mechanisms: first, PEI competitively bound Cu from anionic Cu-citrate complex along with the release of citrate ligands; second, protonated PEI binds anionic citrate to form a PEI–citrate complex and then excess carboxylic groups in PEI-citrate complex could bind additional Cu.

Therefore, the aim of this study is to develop a new amine decorated adsorbent possessing high capacity to HMIs regardless of the presence of OAs or not, and futher explore the interaction mechanisms between HMIs, OAs and the adsorbent. In fact, under weakly acidic solution pH, neutral and protonated amine groups are coexistent in the adsorbent^23^. Moreover, various species of HMIs and OAs in the aqueous phase could also convert each other in dynamic equilibrium during the whole adsorption. We hypothesize that HMIs adsorption by amine adsorbents in HMIs-OAs bi-solutes systems is driven by hybrid mechanisms of both coordination and electrostatic attraction, in which HMIs in cationic or neutral species coordinate with neutral amine sites and anionic complex species directly interact with protonated amine sites.

In this work, a multi-amines decorated resin (PAMD) was newly synthesized by reaction of tetraethylenepentamine and polystyrene-methacrylate resin beads which have high ester content and good mechanical strength. Copper (soluble Cu) and citric acid (CA) were chosen as model pollutants. Cu adsorption in the absence or presence of CA was comparably studied for PAMD and other commercially available resins. To test the hypotheses, the influence of CA in preloaded and coexisting systems on Cu adsorption was evaluated, and the equilibrium distributions of solute species during whole adsorption were qualitatively investigated. Moreover, Electrospray ionization mass spectra (ESI-MS) of the aqueous phase, Fourier Transform Infrared Spectra (FTIR), X-ray Photoelectron Spectra (XPS) and X-ray absorption near edge structure spectra (XANES) of the resin phase were used to further examine the mechanisms of Cu adsorption in the absence and presence of CA.

## Materials and Methods

### Materials

The synthesis route of PAMD is shown in Supplementary Scheme S1. The properties of PAMD and four commercial resins (S984, D113, D001 and D201) are listed in Supplementary Table S1. Cupric nitrate, citric acid, nitric acid, hydrochloric acid, sodium hydroxide, calcium nitrate, potassium phosphate, sodium chloride, sodium sulfate and amine reagent were all reagents of analytical grade purchased from Sinopharm Chemical Reagent Co., Ltd. (Shanghai, P. R. China). All solutions were prepared using ultrapure water produced by a Millipore-Q system (Millipore Synergy, USA). As shown in Supplementary Figure S1, the zeta potential of PAMD was positive at pH-value between 2.0 to 7.0, indicating that amine groups of PAMD were partly protonated.

### Static adsorption experiments

Static batch adsorption experiments were carried out in triplicate by mixing 25 mg dry resin with 100 mL solutions containing Cu and/ or CA with a series of initial concentrations into conical flasks. The initial pH-values of solutions were adjusted to 3.0–7.0 using 1M HNO_3_ and NaOH. The flasks were sealed and continuously agitated at 160 rpm for 48 h under 303K in an incubator shaker. After equilibrium, samples were extracted from the solutions for analysis.

For CA preloaded tests, fresh resin was firstly mixed with CA solution, then filtered after CA adsorption equilibrium, and then added into Cu solution to reach equilibrium again. In terms of kinetic studies, 500 mg PAMD was added in 1 L single solute or bi-solutes solution with various CA/ Cu molar ratios. Samples were collected at pre-set time intervals. Additionally, the effect of coexisting ions on Cu adsorption in single solute or bi-solutes systems was determined using the typical salt (NaCl, Ca(NO_3_)_2_, Na_2_SO_4_ or K_3_PO_4_) as a background component of the mixture solutions.

### Dynamic adsorption

The dynamic adsorption experiments were carried out in a water-jacketed glass column (Φ 10 × 240 mm) with 500 mg dry fresh resin and the wet bed volume (*BV*) was 2.0 mL. Each solution used for this study was adjusted to initial pH-value of 4.0 and pumped into the column in a downward flow direction using a peristaltic pump at the flow rate of 5 BV/h under 303 K. Regeneration of the exhausted resin bed in dynamic study was performed as downward pumping 2 M HCl solution in 1 BV/h at room temperature until no Cu or CA was detected in the effluent. Aliquots of the effluent were taken at regular time intervals to determine the concentrations of pollutants in the effluent (*C*_*t*_), *t* was the operation time of the column. The saturation of the column was set at *C*_*t*_*/C*_*0*_ = 0.95.

### Analytical procedures

#### Detection methods

The concentration of soluble Cu in aqueous phase was measured by atomic absorption spectrophotometer (AAS, THERMO, USA). The concentration of CA was determined using ion chromatography (Dionex 1000) with an IonPac AS11-HC (4 mm × 250 mm) column. 40 mM KOH solution was used as eluent at a flow rate of 1.0 mL/min. The retention time of CA was around 8.17 min.

#### Adsorption equations and models

The adsorption amount at equilibrium (*Q*_*e*_ or *Q*, mmol/g) and the enhancement rate of adsorption amount in bi-solutes system compared with that in single solute system (*E*_*r*_) are calculated using Eq.1 and Eq.2. Pseudo-first-order model and pseudo-second-order kinetic model are commonly used to describe elementary reversible adsorptive reactions on solid phases in aqueous media^24^,^25^. The models can be expressed as Eq. 3 and Eq.4.


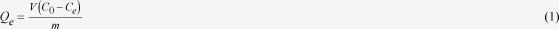



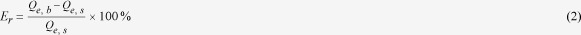



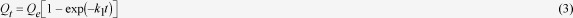



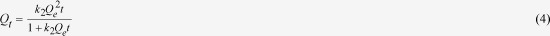
where, *C*_*0*_ and *C*_*e*_ are the initial and equilibrium concentration (mmol/L), and *m* is the mass of resin (g), *V* is the volume of solution (L). *Q*_*t*_ is adsorption amount over time period *t* (mmol/g). *k*_*1*_ and *k*_*2*_ (g/mmol/min) are the constants associated with the adsorption rate in the corresponding equation. *h = k*_*2*_*Q*_*e*_^2^ is defined as the initial adsorption rate constant.

### Characterization techniques

The speciation information in the aqueous phase was analyzed by theoretical calculation from Visual MINTEQ (ver. 3.0, USA) and ESI-MS (LCQ Fleet ESI Mass Spectrometer, USA). Resin samples exposed to single solute solution (containing Cu or CA) and bi-solutes solution (containing both Cu and CA) are named as PAMD + Cu, PAMD + CA, PAMD + Cu + CA, respectively. The initial concentration of each pollutant in each system was 2 mmol/L. Solid-state IR spectra (KBr pellets) in the region of 4000–400 cm^−1^ were recorded on a VERTEX 80 v Fourier transform infrared spectrometer (Bruker, Germany). XPS results were obtained from an X-ray photoelectron spectrometer (ESCALAB-2, Great Britain) and analyzed using the XPSPEAK41 software. Binding energies in the XPS spectra refer to the neutral C1s peak at 284.6 eV to compensate for the surface charging effects and systematic errors^26^. XANES measurements were performed at the X-ray absorption station of the 1W1B beamline at the Beijing Synchrotron Radiation Facility. During the experiment, the storage ring was operated at 2.5 GeV, with a beam current of approximately 200 mA. The absolute energy position was calibrated using a Cu metal foil, and a Si (111) double-crystal monochromator was used to monochromatize the radiation. To suppress the unwanted high-order harmonics, the parallelism of the two crystals in the monochromator was adjusted to mistune the incident beam by 30%. The incident beam intensities were monitored and recorded using a nitrogen gas-flow ionization chamber. Three reference compounds comparing with resin samples were Cu(NO_3_)_2_, cupric citrate (Cu_2_C_6_H_4_O_7_·2.5H_2_O, CAS: 866-82-0, Sinopharm Chemical Reagent Co., Ltd., Shanghai, China) and Cu + Amine (dilute mixture solution of Cu and tetraethylenepentamine). Resin samples and cupric citrate (s) were measured in the transmission mode, while aqueous Cu(NO_3_)_2_ and Cu + Amine were measured in the fluorescence mode. All of them were scanned three times and averaged.

## Results and discussion

### Complexation characteristics in the aqueous phase

CA bears three carboxyl and one hydroxyl groups and shows great complexation affinities for many heavy metal ions like Cu ion^27^. Knowledge of Cu speciation is essential to interpret the environmental behavior of Cu in solid/ aqueous phase. Cu species was investigated by theoretical calculation and ESI-MS (details were shown in Supplementary Figure S2 and S3). Consistent with results in earlier reports^28^, [Cu-CA] complexes including CuHL^0^, CuL^−^ and Cu_2_L_2_^2−^ (L means CA ligand) were formed at pH-values between 2.0 to 7.0 in bi-solutes systems, accounting for 10 to 90% of total Cu. With the increase of CA concentration at pH 4.0, the proportions of complex species increased and Cu^2 + ^(refers to ligand-free Cu ion) decreased (see Supplementary Table S3). Furthermore, the chemical structures of complex species were explored using the Density-Functional-Theory (DFT) as shown in Supplementary Figure S4.

### Effect of CA on the adsorption of Cu

Four commercial resins (D001, D113, D201 and S984) were chosen to compare with PAMD. D001 and D113 are cation exchange resins containing sulfonic (−SO_3_H) and carboxyl (−COOH) groups, respectively, while D201 is an anion exchange resin bearing quaternary amine (−NR_3_^ + ^)^29^,^30^,^31^. S984 characterized by polyamine groups has similar structure to that of PAMD^32^. Cu adsorption of the five resins with different CA concentrations was investigated (see Supplementary Figure S5). Along with the increase of CA concentrations, the adsorption amount of Cu onto both D001 and D113 markedly decreased by 73% and 80%. Since most of Cu was present in forms of anionic [Cu-CA] complex species, electrostatic repulsion occurred and hindered the contact of Cu with the resin and their interaction^33^. Oppositely, Cu adsorption onto D201, S984 and PAMD increased in the presence of CA. The enhancement was probably related to large adsorption of CA onto these resins (see Supplementary Figure S6). Since Cu was hardly adsorbed onto D201 from Cu solution without CA, Cu adsorption capacities of D201 in bi-solute systems were far lower than PAMD. In addition, the adsorption enhancement rate (*E*_*r*_) of Cu obtained for S984 (102%) was much smaller than that obtained for PAMD (180%). This difference can be explained by different amount of active amine sites. Cu adsorption capacity of PAMD finally became the highest (4.25 mmol/g) and exceeded all the tested commercial resins by 1.8 to 7 folds in the same bi-solutes system. These observations confirmed the superior performance of PAMD for highly efficient removal of both Cu and CA from wastewaters.

Considering that the pH values of real wastewaters varied generally between 3 and 7, adsorption was investigated at various pH values (see Supplementary Figure S7). The results indicated similar trends and the adsorption capacity of PAMD for Cu and CA was both pH-dependent and maximum at pH 4.0, which was probably attributed to the best match of distributions of solute species and amine site species under this pH condition. With the increase of solution pH, anionic species increased but positive amine sites decreased.

### Mechanisms for the enhancement effect

In consideration of the various Cu species coexisting in bi-solutes systems, the observed enhancement in our work should be driven by hybrid mechanisms such as site-bridging effect, anionic complex adsorption or both them. Therefore, to illustrate the detailed interaction mechanisms, the influence of CA preloaded and coexisting on Cu adsorption and Cu speciation in aqueous/ resin phase during adsorption were discussed.

#### Comparison of preloaded and coexisting systems

**Figure 1:**
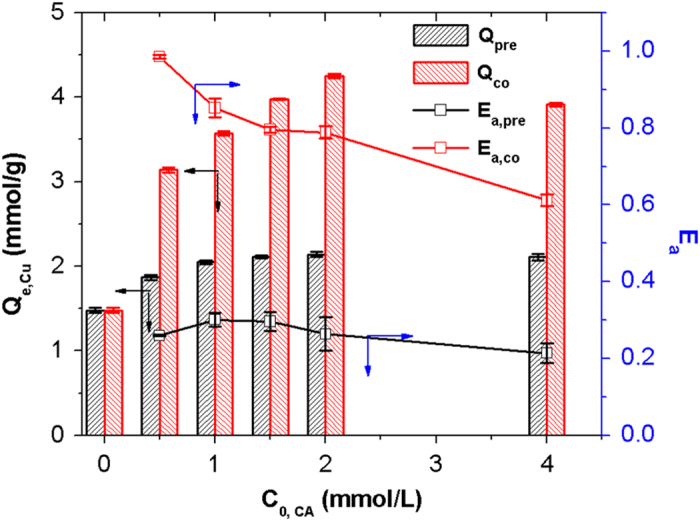
Adsorption of Cu onto PAMD and Ea values in preloaded and coexisting systems. Resin dosage: 25 mg (dry weight basis), 100 mL; Initial concentration of Cu (C_0_, Cu) was fixed at 2.0 mmol/L, initial concentration of CA (C_0_, CA) ranged from 0 to 4.0 mmol/L; Initial pH was 4.0; under 303 K, 48 h.

Cu adsorption onto PAMD in preloaded system (where CA was adsorbed onto the resin first) and coexisting system (where CA adsorption was concurrent with Cu adsorption) was studied at the same CA initial concentrations. Cu adsorption onto PAMD also increased with the increase of CA preloaded amount (Fig. 1). This proved that, to a certain degree, CA in the resin phase could provide new active sites (e.g. carboxyl and hydroxyl groups) which played a positive role of the role of bridge between Cu and the resin to form resin-CA-Cu ternary complex^34^. However, Cu adsorption of PAMD in coexisting system was much higher than that in preloaded system at the same CA initial concentration. A more direct comparison of Cu adsorption enhancement in the two systems can be made by measuring CA adsorption-normalized enhancement amount (*E*_*a*_) which is calculated as Eq. 5 below.


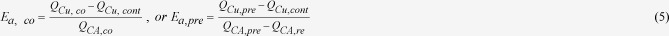


where, the abbreviations of *co, pre, cont* refer to the coexisting system, CA preloaded system and control system where Cu was applied alone, respectively. *Q*_*CA,re*_ is the release amount of CA in Cu solution at equilibrium in preloaded systems.

The value of *E*_*a, co*_ was three times higher than that of *E*_*a pre*_, suggesting that the enhancement of Cu adsorption in the two systems was caused by two different mechanisms. Moreover, the release amount of CA during Cu adsorption in preloaded system was considerable (up to 18% of CA preloaded amount) and linearly increased with the increase of CA preloaded amount (Supplementary Figure S8). In contrast, there was negligible release of CA in water. The results suggested that the binding affinity between CA and Cu was higher than affinity between CA and the resin. Therefore, CA site-bridging effect was not the main reason for the remarkable enhancement of Cu adsorption in the presence of CA.

#### Kinetic processes

**Figure 2:**
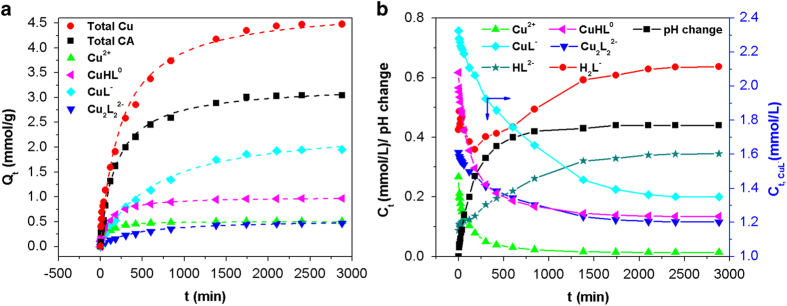
Kinetic curves for adsorption of total Cu, total CA and major species (**A**), and variations of major species concentrations and solution pH (**B**). Adsorption data are fitted by second-order kinetic model (dash lines). pH change was calculated as the difference between the final pH-value and the initial pH-value. Resin dosage: 500 mg (dry weight basis), 1000 mL; Initial concentrations of Cu and CA were both 4.0 mmol/L (the high concentration is set for enlarging phenomena); Initial pH was 4.0; Under 303 K, 48 h.

According to the above discussions, [Cu-CA] complex should be directly adsorbed with a high affinity. Kinetic adsorption in a bi-solutes system with equimolar ratio of CA and Cu was shown in Fig. 2. The adsorption of total Cu and total CA was better fitted by pseudo-second-order model than pseudo-first-order model according to the calculated correlation coefficients (*R*^*2*^) (Fig. 2A and Table 1), thereby suggesting that the overall process was controlled by chemisorption^35^. Further, the concentrations of aqueous species at *t* time were calculated using Visual MINTEQ based on the concentrations of total CA, total Cu and solution pH value^36^,^37^. Figure 2B describes the concentration variations of major species and pH change during the whole adsorption period. The initial concentrations of them were in the order of CuL^−^ (2.33 mmol/L)» CuHL^0^ (0.61 mmol/L) > H_2_L^−^ (0.42 mmol/L) > Cu_2_L_2_^2−^ (0.35 mmol/L) > Cu^2 + ^(0.26 mmol/L) > HL^2−^ (0.09 mmol/L). As expected, the concentrations of the four Cu species dropped with adsorption proceeding. However, the concentrations of the two Cu-free CA species showed an opposite trend, gradually rising to 0.63 mmol/L for H_2_L^−^ and 0.34 mmol/L for HL^2−^. Therefore, the preferred adsorbate species for PAMD in bi-solutes system were Cu^2 + ^ and [Cu-CA] complexes instead of Cu-free CA species, which also reinforces our earlier conclusion that site-bridging effect is not the mechanism for the large enhancement in Cu adsorption. In addition, the increase of solution pH during adsorption was resulted from OH^−^ release during the protonation of amine groups^38^.

The adsorption of four adsorbate species (Cu^2 + ^, CuHL^0^, CuL^−^ and Cu_2_L_2_^2−^) was also fitted by kinetic models. The adsorption of Cu^2 + ^ and CuHL^0^ was much faster than that of CuL^−^ and Cu_2_L_2_^2−^ and reached equilibrium around 300, 600, 1400 and 2400 min, respectively (Fig. 2A). The values of *k*_*2*_ and *h* for Cu^2 + ^ and CuHL^0^ were one order of magnitude higher than those for CuL^−^ and Cu_2_L_2_^2−^ (Table 1). The result implies two kinds of mechanisms: both Cu^2 + ^and CuHL^0^ interacted with neutral amine sites through coordination^39^, while CuL^−^ and Cu_2_L_2_^2−^ interacted with protonated amine sites via electrostatic attraction^20^. Considering Cu and CA in all complex species was at 1:1 mole ratio, in theory, *Q*_*CA, b*_ should be equal to the difference between *Q*_*Cu*,*b*_ and *Q*_*Cu*_^*2*+^. In contrast, it was found that the value of *Q*_*CA, b*_ was far less than the difference (Table 1). Therefore, Cu in at least one [Cu-CA] complex species was adsorbed along with CA release. Because of electrostatic repulsion, it was hard to occur that Cu in anionic species (CuL^−^ and Cu_2_L_2_^2−^) bonded to protonated amine sites simultaneously with the release of CA ligand. Alternatively, the decomplexation of neutral complex (CuHL^0^) was probably concurrent with the coordination between Cu and neutral amine sites, since the complex formation constant of [Cu-amine] complex is greater than that of CuHL^0^ 21,40. This is also evidenced by the gradual increase of HL^2−^ and H_2_L^−^ during the adsorption period. Moreover, the sum of *Q*_*Cu*_^*2*+^ and *Q*_*CuHL*_^*0*^ was approximately equal to the difference between *Q*_*Cu, b*_ and *Q*_*CA, b*_ (Table 1, Eq. 6), thereby confirming the mechanism that Cu in CuHL^0^ was adsorbed via coordination with neutral amine sites along with the release of CA. The adsorption kinetics in bi-solutes systems with excess Cu and excess CA was also investigated and successfully confirmed the aforementioned behaviors and mechanisms (see Supplementary Figure S9 and S10).


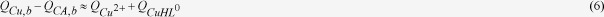


#### Quantitative examination of dual-sites interaction

According to the proposed mechanisms, the maximum Cu adsorption capacity of PAMD in bi-solutes system should be dependent on the amounts of both coordination sites (neutral amines, Site I) and electrostatic attraction sites (protonated amines, Site II). To examine the dual-sites interaction, a series of dynamic column experiments were conducted (Supplementary Figure S13A and Text S1). The saturated adsorption capacities of Cu and CA obtained in their single solute systems were 1.77 mmol/g and 3.28 mmol/g, corresponding to the available amounts of Site I and Site II, respectively. Meanwhile, the saturated adsorption capacity of Cu obtained in bi-solutes system (*Q*_*S,Cu,b*_) was 5.07 mmol/g, which was approximately equal to the sum of amounts of Site I and Site II (Eq. 7).


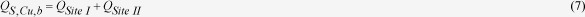


#### Confirmation with FTIR, XPS and XANES

**Figure 3:**
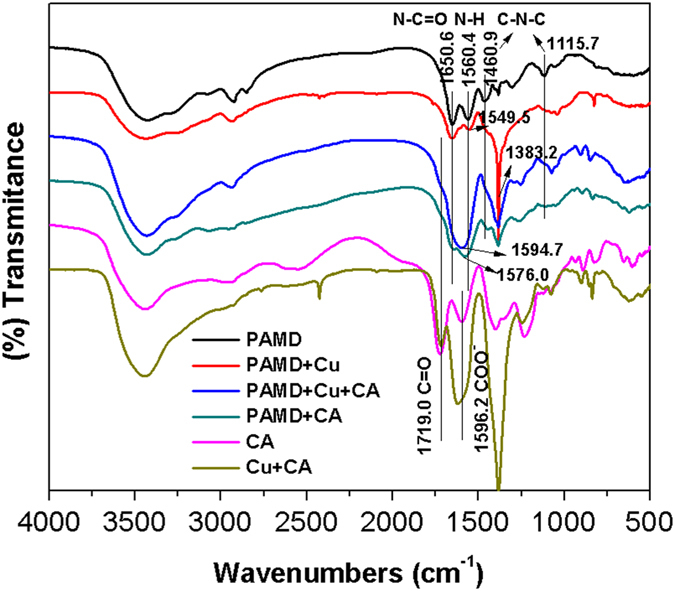
FTIR spectra of solid-state solutes and resin samples. Solid-state solutes (CA and Cu + CA) were obtained by freeze-drying the corresponding solutions without adsorption. Vertical lines indicate peaks of interest.

The FTIR spectra of two solid-state solutes (CA and Cu + CA) and four resin samples (PAMD, PAMD + Cu, PAMD + Cu + CA, PAMD + CA) were recorded (Fig. 3). Two adjacent peaks at 1719.0 cm^−1^ and 1596.2 cm^−1^ in the spectra of CA were assigned to the stretching vibrations of C = O and COO^−^ in CA species, respectively^41^. However, in the spectrum of Cu + CA, an obvious shift (~25 cm^−1^) of the stretching vibration of COO^−^ (showing at 1621.1 cm^−1^) was observed while the peak of C = O stretching vibration remained identical, thereby confirming the complexation between Cu and carboxyl of CA. In comparison of the spectra of PAMD and PAMD + Cu, the bending vibration of –NH– showed an obvious shift (PAMD: 1560.4 cm^−1^; PAMD + Cu: 1549.5 cm^−1^) and the vibration of C-N-C band (1460.9 and 1115.7 cm^−1^) disappeared, while the stretching vibration of N-C = O at 1650.6 cm^−1^ remained unchanged^35^,^42^. It can be concluded that amine groups instead of carbonyl in the resin coordinated with Cu in Cu single solute system. The new sharp peak at 1383.2 cm^−1^ in PAMD + Cu spectrum was attributed to the stretching vibration of NO_3_^−^ which was adsorbed for charge balance along with the coordination of Cu^2 + ^and amine^43^. Based on the above analyses, the vibrations of C = O band of CA species and N-C = O groups of resin can be recognized in the spectra of PAMD + CA and PAMD + Cu + CA, which remained the same with those of CA, PAMD and PAMD + Cu. However, NO_3_^−^ vibration was hardly detected in PAMD + Cu + CA spectrum, consistent with the decrease of Cu^2 + ^adsorption. Unfortunately, it is hard to gain more insight because the vibrations of –NH– and -COO^−^ merged into a broad band at 1594.7 cm^−1^ for PAMD + Cu + CA and 1576.0 cm^−1^ for PAMD + CA.

**Figure 4:**
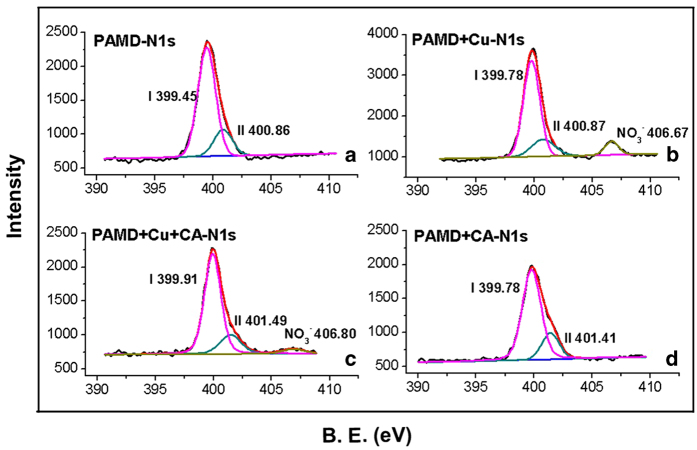
N1s XPS spectra of PAMD (**A**), PAMD + Cu (**B**), PAMD + Cu + CA (**C**) and PAMD + CA (**D**).

XPS characterization of the resin samples was performed to further investigate the change of chemical structure during adsorption. Wide scans shows characteristic peaks of C1s (284 eV), N1s (400 eV) and O1s (531 eV) in each sample and extra Cu2p (932–952 eV) in PAMD + Cu and PAMD + Cu + CA (see Supplementary Figure S11A-D)^44^. The high-resolution spectra N1s are shown in Fig. 4. Spectrum of N1s in PAMD is deconvoluted into two different component peaks at the binding energy (B.E.) of 399.45 and 400.86 eV, corresponding to the nitrogen in the neutral amines (-NH_2_ or -NH-, Site I) and protonated amines (-NH_3_^ + ^or –NH_2_^ + ^-, Site II)^45^,^46^, respectively. For PAMD + Cu, the peak for Site I shifted to 399.78 eV without obvious change of peak for Site II, showing the coordination between Cu^2 + ^and Site I, in which N atom donates a pair of free electrons to form the coordination bond N-Cu, causing the reduction of electron cloud density. There was also a new peak at 406.67 eV attributed to nitrogen in NO_3_^−^ 47. In contrast, for PAMD + Cu + CA, both the peak of Site I and Site II showed obvious shifts to 399.91 and 401.49 eV, respectively. The shifts suggest that both the Site I and Site II participated in the adsorption of Cu in bi-solutes system. Consistent with the FTIR results, NO_3_^−^ peak at around 406.80 eV was very weak in PAMD + Cu + CA spectrum. In addition, there was no obvious change in O1s spectrum of each sample (see Supplementary Figure S11E-F) possibly because O1s spectra represented the mixture status of O in amide, carboxyl, hydroxyl and NO_3_^−^ groups. It was hard to distinguish them from XPS spectra.

**Figure 5:**
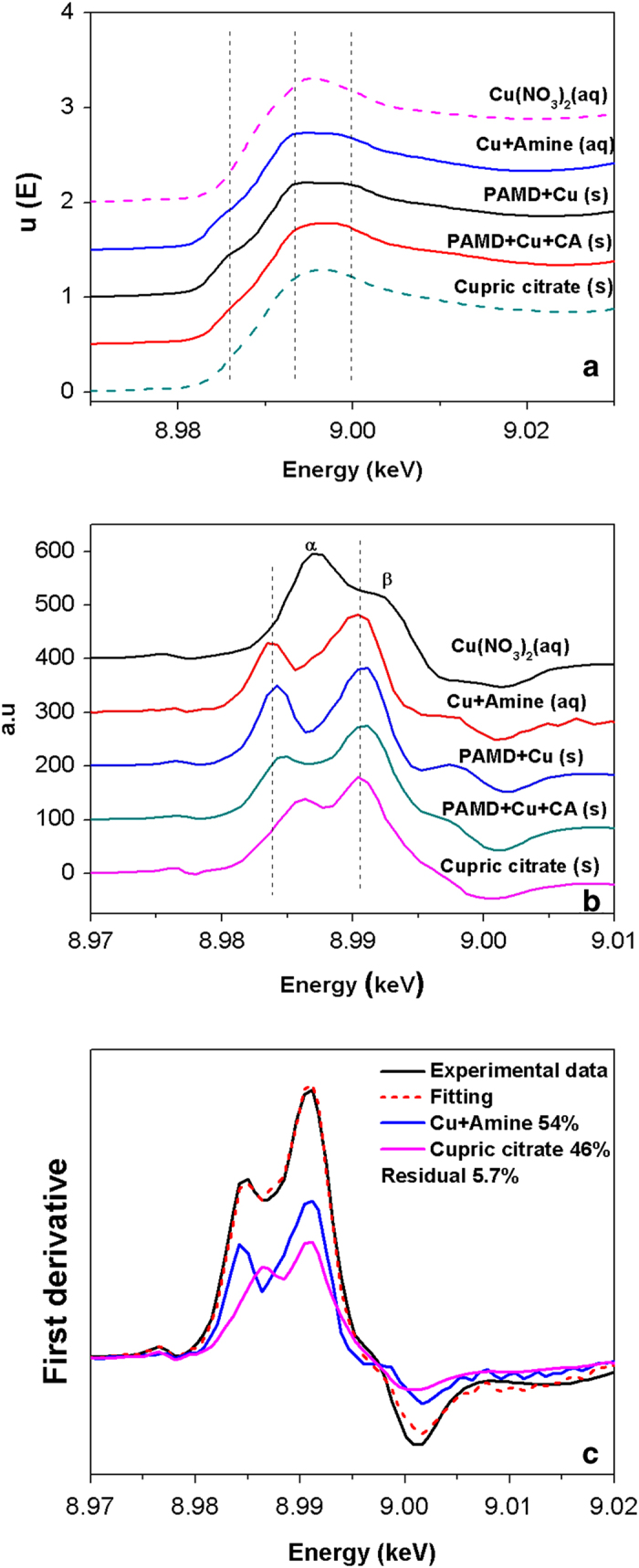
Normalized XANES spectra of resin samples and reference compounds (**A**), and the corresponding first derivatives (**B**) and LCF result (**C**) for first derivative spectrum of PAMD + Cu + CA (Two weighted components are displayed together). All the data were processed and analyzed using WinXAS 3.1.

XANES spectra of resin samples in the single-solute and bi-solutes system were also measured and compared with those of three reference compounds (Cu(NO_3_)_2_, Cu + Amine and cupric citrate). The similarity of XANES spectra of PAMD + Cu and Cu + Amine directly demonstrate the interaction between Cu and amine sites of PAMD (Fig. 5A). The shoulder peak at 8.986 keV was caused by 1s to 4p transitions of Cu^2 + ^, and it reveals a tetragonal distortion of Cu^2 + ^ configuration in PAMD + Cu^48^. As for PAMD + Cu + CA, the intensity of peaks at 8.986 and 9.000 keV weakened when compared with those in PAMD + Cu (Fig. 5A). The difference between the two samples indicated that the coordination environment of Cu on PAMD was significantly affected by CA. To better understand the XANES features, the first derivatives of these spectra are also compared (Fig. 5B). The first derivative spectra for all resin samples and reference compounds shows two different inflections corresponding to the derivative peaks α and β. The peak β represented the main absorption transition (1s → continuum). The peak α was influenced by the degree of bond covalency and the degree of local structural disorder^49^. For the aqueous Cu(NO_3_)_2_ solution, the intensity of peak α was greater than that of peak β. When Cu species was adsorbed from water to resin, the intensity of peak α became less intense, suggesting that water molecules in the tetragonal plane around Cu were partially replaced by the organic ligands from PAMD or/and CA^48^,^50^. Furthermore, least-square linear combination fitting (LCF) technique was successfully applied to distinguish and quantify the main components of PAMD + Cu + CA using Cu + Amine and cupric citrate spectra as fitting standards, resulting in about 54% Cu + Amine and 46% cupric citrate, with a residual of 5.7% (Fig. 5C). Since there is two unit of Cu in per unit of the cupric citrate standard, the ratio of Cu bonded to amine and Cu bonded to CA was calculated as 0.587. Importantly, the ratio is directly associated with the proportions of Cu adsorption driven by two interaction mechanisms (coordination and electrostatic attraction) in bi-solutes systems, and very much agreed with the results of kinetic studies (see Table 1).

#### Mechanism simulation

**Figure 6:**
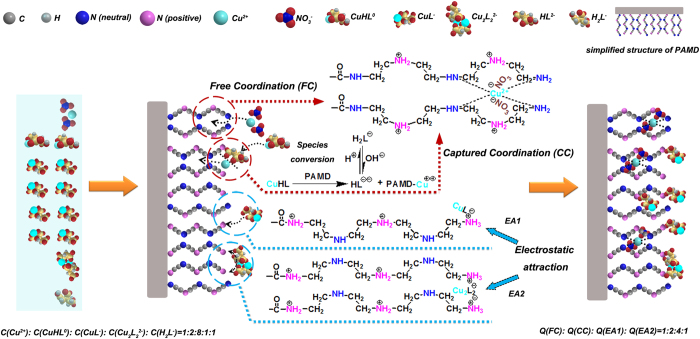
Dual-sites interaction mechanism in bi-solutes systems.

The above discussions suggest that the large enhancement of Cu adsorption onto PAMD in bi-solutes systems was attributed to the switch of mechanism from single-site to dual-sites interaction. In single solute system, all Cu was Cu^2 + ^ which could only interact with the Site I through *Free coordination (FC)*. In contrast, in a bi-solutes system with equimolar ratio of Cu and CA, Cu species mainly consisted of Cu^2 + ^, CuHL^0^, CuL^−^ and Cu_2_L_2_^2−^at an approximate concentration ratio of 1:2:8:1 (according to the species distribution calculation in kinetic studies). Besides playing a role in *FC*, Site I also coordinated with Cu in CuHL^0^ species while HL^2−^ ligand was released to the aqueous phase. The process was defined as *Captured Coordination (CC)*. More importantly, abundant anionic Cu species (CuL^−^ and Cu_2_L_2_^2−^) were adsorbed onto Site II via electrostatic attraction at the molar ratio of 1:1 and 1:2 respectively, which was defined as *EA 1* and *EA 2*. Finally, Cu^2 + ^, CuHL^0^, CuL^−^ and Cu_2_L_2_^2−^ species in resin-phase was found in an approximate ratio of 1:2:4:1 (see Table 1), corresponding to the mechanism contribution ratio of *FC, CC, EA1* and *EA2*. The proposed dual-sites interaction mechanism for the large adsorption of Cu in bi-solutes system could be quantitatively illustrated by Fig. 6 and Eq. 8–11.


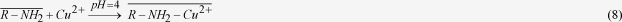



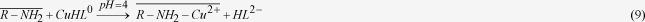



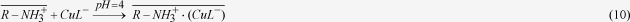



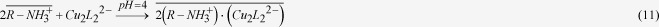


### Assessments of practical application

#### Effect of common coexisting ions

Abundant common ions such as Na^ + ^, Ca^2 + ^, Cl^−^, NO_3_^−^, SO_4_^2−^, and PO_4_^3−^ are often present in wastewaters^51^,^52^,^53^. Therefore, it is important to investigate the influence of these coexisting cations and anions on Cu adsorption by PAMD with or without CA. For single solute system, Cu adsorption capacity of PAMD showed a small increase (maximum ~15.4%) in the presence of all salts (see Supplementary Figure S12A). Since Na^+^, K^+^ and Ca^2+^ have very low affinity to bond with amine sites according to HSAB theory, the increase probably resulted from the electrostatic shielding effect of anions^54^,^55^. In contrast, Cu adsorption on cation exchange resins D001 and D113 was much suppressed by salts because of competitive exchange of these coexisting cations. For bi-solutes system, a slight decrease of Cu adsorption by PAMD occurred in the presence of all salts, especially for highly charged anions. However, anionic [Cu-CA] complex species showed higher affinities than Cl^−^, NO_3_^−^, SO_4_^2−^, and PO_4_^3−^ to interact with positive amine sites (see Supplementary Figure S12B)^56^. Similar results were obtained for S984 while Cu adsorption of D201 decreased more obviously. For the two cation exchange resins, both the competition of common cations and complexation of CA led to the very low adsorption of Cu. Overall, PAMD showed the best anti-interference ability and the largest Cu capacity among all tested resins from mixture wastewaters, indicative of the possible application of PAMD in real wastewaters.

#### Dynamic adsorption and regeneration

Typical breakthrough curves of Cu in single solute and bi-solutes system were obtained (see Supplementary Figure S13A). Cu concentrations detected in the effluent were below 1.3 mg/L before 60 BV for single solute system and 285 BV for bi-solutes system, respectively, which met the standard of National Primary Drinking Water Regulations (NPDWRs) from US EPA^57^. 2 M HCl was used to regenerate the exhausted resin. Highly concentrated protons replaced Cu and totally protonated both CA and amine groups. Over 99% of the pollutants were recovered by applying 20 BV HCl and the regeneration solution finally contained 4051 mg/L Cu (63.3 mmol/L) and 10743 mg/L CA (55.9 mmol/L), which could be reused for production processes (Supplementary Figure S13B). Then, PAMD were also regenerated and reused. Furthermore, PAMD had the stable and excellent performance after five cycles of column adsorption and desorption procedures (see Supplementary Figure S14).

#### Applications for other similar wastewater systems

To further prove the universality of large enhancement effect in our work, PAMD was also tested in other similar systems involving HMIs (Cu/ Ni/ Zn) and OAs (tartaric acid (TA)/ citric acid (CA)/ oxalic acid (XA)/ ethylenediamine tetraacetic acid (EDTA)). OAs consistently enhanced the adsorption of HMIs onto PAMD by 99.6% ~ 230.1% (see Supplementary Figure S15), which implies a great application potential of this newly synthesized resin.

## Conclusions

The new multi-amines decorated resin PAMD showed highly efficient removal of Cu from wastewater containing CA, and is recyclable and stable. Cu adsorption capacity of PAMD was enhanced from 1.77 mmol/g in the absence of CA to 5.07 mmol/g in the presence of CA. The large enhancement was driven by the mechanism change from single-site to dual-sites interaction in which cationic or neutral Cu species (Cu^2 + ^and CuHL^0^) coordinated with neutral amine sites and anionic complex species (CuL^−^ and Cu_2_L_2_^2−^) directly interacted with protonated amine sites via electrostatic attraction. The proportion of the four Cu species adsorbed was approximately 1:2:4:1 for equimolar bi-solutes systems. Additionally, the superior performance of PAMD was also found in other similar systems containing HMIs and OAs. Thus, PAMD has a very attractive potential of the practical application for treating this kind of wastewaters. Moreover, the new insight in the interaction between [Cu-CA] bi-pollutants and adsorbents with amines has important implications for the surface functionalization of materials for environmental application and the fate of HMIs in environmental media containing both OAs and amines.

## Additional Information

**How to cite this article**: Ling, C. *et al*. Citric Acid Enhanced Copper Removal by a Novel Multi-amines Decorated Resin. *Sci. Rep*. **5**, 9944; doi: 10.1038/srep09944 (2015).

## Supplementary Material

Supplementary Information

## Figures and Tables

**Table 1:** 

Targets		Pseudo-first-order model			Pseudo-second-order model			
	a*Q*_*e, exp*_	b*Q*_*e, fit*_	*k*_*1*_	*R*^*2*^	b*Q*_*e, fit*_	*k*_*2*_	*h*	*R*^*2*^
Total Cu	c4.37	4.28	3.2E^−3^	0.971	4.72	8.81E^−4^	1.96E^−2^	0.988
Total CA	c2.97	2.84	4.2E^−3^	0.984	3.18	1.71E^−3^	1.73E^−2^	0.998
Cu^2+^	cf0.51	0.48	1.6E^−2^	0.945	0.51	4.25E^−2^	1.11E^−2^	0.988
CuHL^0^	c0.97	0.92	7.6E^−3^	0.977	1.00	1.04E^−2^	1.04E^−2^	0.996
CuL^−^	c1.95	1.97	1.6E^−3^	0.990	2.45	6.51E^−4^	3.91E^−3^	0.993
Cu_2_L_2_^2−^	c0.47	0.46	2.1E^−3^	0.983	0.55	4.44E^−3^	1.34E^−3^	0.990

^a^*Q*_*e, exp*_ and ^b^*Q*_*e, fi*t_ are adsorption amounts at equilibrium obtained from experiments and kinetic model fitting. ^c^In the text, for briefness, *Q*_*Cu, b*_ and *Q*_*CA, b*_ are the total equilibrium adsorption amounts of Cu and CA in bi-solute systems, while *Q*_*Cu*_^2+^, Q_*CuHL*_^*0*^, Q_*CuL*_^−^, and 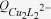 are the equilibrium adsorption amounts of the corresponding adsorbate species. The value of (Q_*Cu*_^2+^+Q_*CuHL*_^*0*^)/(Q_*CuL*_^−^+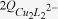) is 0.51.
