# Supplementary material for: Citric Acid Enhanced Copper Removal by a Novel Multi-amines Decorated Resin
Source: Sci Rep. 2015 May 12;5:9944. doi: 10.1038/srep09944 (PMC4649996; doi:10.1038/srep09944)
Supplement: Supplementary Information [file srep9944-s1.pdf]

Supplementary Information

Citric Acid Enhanced Copper Removal by a Novel Multi-amines Decorated Resin

Chen Ling[1], Fuqiang Liu[*, 1], Zhiguo Pei[*, 2], Xiaopeng Zhang[1], Mengmeng Wei[1], Yanhong Zhang[1], Lirong Zheng[4], Jing Zhang[4], Aimin Li[1], Baoshan Xing[4]

[1]State Key Laboratory of Pollution Control and Resource Reuse, School of the Environment, Nanjing University, Nanjing 210023, P. R. China

[2]State Key Laboratory of Environmental Chemistry and Ecotoxicology, Research Center for Eco-Environmental Sciences, Chinese Academy of Sciences, Beijing 100085, China

[3]Beijing Synchrotron Radiation Laboratory, Institute of High Energy Physics, Chinese Academy of Sciences, Beijing 100049, China

[4]Stockbridge School of Agriculture, University of Massachusetts, Amherst, Massachusetts 01003, United States

[*]Corresponding authors:

Tel: +86 139 1387 1032, Fax: +86 25 89680377. Email address:jogia@163.com (F. L), peizg@rcees.ac.cn (Z. P)

Contents

Part I. Adsorbents

Synthesis of PAMD (Scheme S1)

Physicochemical characteristics of adsorbents in this work (Table S1)

Zeta potential measurement of PAMD (Figure S1)

Part II. Investigation of complexation between Cu and CA in the aqueous solutions

Calculation of species distribution (Table S2, Figure S2 and Table S3)

Examination with ESI-MS (Figure S3)

Complexation structure and DFT optimization (Figure S4, Table S4)

Part III. Supplementary experiments

Resin Comparison (Figure S5, Figure S6 and Figure S7)

CA release from PAMD in preloaded system (Figure S8)

Kinetic studies for systems with excess CA or excess Cu (Figure S9, Table S5, Figure S10, and Table S6)

XPS wide scan and O1s spectra of resin samples (Figure S11)

Effect of common ions (Figure S12)

Fixed-bed dynamic adsorption and resin regeneration (Text S1, Figure S13 and Figure S14)

The application of PAMD in other similar systems (Figure S15)

Part I. Adsorbents

Synthesis of PAMD

PAMD was prepared as following steps: 10 g of polystyrene-methacrylate resin beads (PAM) were swelled in 150 mL of tetraethylenepentamine for 10h at room temperature. Then shifted them to a 250mL three-necked round bottle flask with gentle stirring, raised the reaction temperature to 393K with an oil bath and kept for 24 h. Finally the resin beads were separated by filtration with a Buchner funnel and repeatedly washed with ultrapure water until neutral pH (7~8), then dried in a vacuum dryer at 313 K to a constant weight. The synthesis routes were shown in Scheme S1.

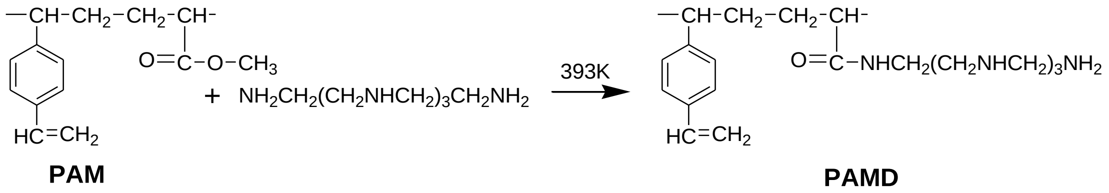

Scheme S1. Synthesis route of PAMD.

Physicochemical characteristics of adsorbents in this work

The physicochemical characteristics of PAMD, D001, D113, S984 and D201 were listed in Table S1.

Table S1. Physicochemical characteristics of adsorbents.

Properties	PAMD	D001	D113	S984	D201
Matrix structure	[a]PS-MA	[b]PS-DVB	PS-MA	PS-MA	PS-DVB
BET surface area (m^2/g)	4.78	15.8	0.39	4.17	16.4
Pore volume (cm^3/g)	0.007	0.102	0.024	0.004	0.095
Average pore diameter (nm)	5.88	28.2	1.81	19.22	26.7
Functional group and content	amine	sulfonic acid	carboxyl	polyamine	quaternary amine
	N:21%	S: 14%	O: 36%	N: 18%	N:8%

[a]PS-MA and [b]PS-DVB are abbreviations of polystyrene methacrylate and polystyrene divinylbenzene, respectively. The information of specific surface area and porous texture of adsorbents was determined by N_2 isotherms data at 77 K using an adsorption analyzer ASAP 2020 (Micromeritics Instrument Co., USA), and calculated with Brunauer-Emmett-Teller (BET) and Dubinin-Radushkevich (DR) methods. The element content of functional group was examined with Germany CHN-O-Rapid elemental analyzer.

Discussion: All the five adsorbents have relative low surface area and small pore volume but high contents of hydrophilic functional groups, suggesting that their adsorption abilities were mostly attributed to surface chemical interactions.

Zeta potential measurement of PAMD

The zeta potential of PAMD was measured by zeta sizer (ZetaPALS, Brookhaven Instruments Ltd., USA). 50 mg PAMD powder was mixed in 500 mL ultrapure water and sonicated for 1 h. The suspension was parted to be adjusted to different pH values (2-12) with dilute NaOH and HNO_3 solution. Each of resin suspension was kept shaking for 24 h before measurement. Each sample was measured 5 times and averaged.

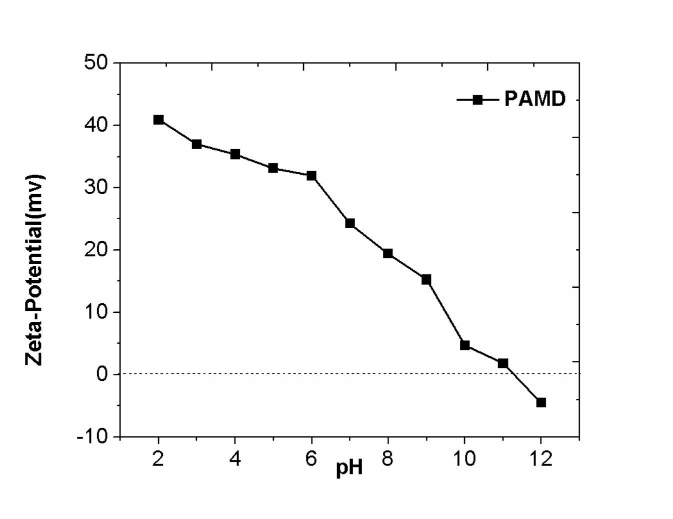

Figure S1. Zeta potentials of PAMD as a function of aqueous pH-values

<u>Discussion</u>: From Figure S1, under wide pH range, the zeta potential of PAMD was positive and gradually decreased in higher pH values, directly proving the existence of large amount of protonated amine sites (–NH_3^+, -NH_2^+-, etc.).

Part II. Investigation of complexation between Cu and CA in the aqueous phase

Calculation of species distribution

The distribution of all possible species has been calculated with Visual MINTEQ software (ver. 3.0, USA)[1,2]. The corresponding logβ constants of the species were listed in Table S2. Although the preparation of Cu solutions and pH adjusting would bring NO_3^- into the aqueous phase, it was found that NO_3^- concentrations (in the range of our tests) showed no influence on aqueous distributions of Cu and CA complex. Figure S2 showed the distribution of major species (the proportion is larger than 5%) under different pH-values.

Table S2. The formation constant (logK_f) of possible species[3, 4].

Species	Reaction	logK_f
H_3L^0	$3H^++L^{3-}=H_3L^0$	14.3
H_2L^-	$2H^++L^{3-}=H_2L^-$	11.2
HL^{2-}	$H^++L^{3-}=HL^{2-}$	6.4
CuH_2L^+	$Cu^{2+}+2H^++L^{3-}=CuH_2L^+$	13.2
$CuHL^0$	$Cu^{2+}+H^++L^{3-}=CuHL^0$	11.0
CuL^-	$Cu^{2+}+L^{3-}=CuL^-$	7.6
Cu_2L^{2-}	$2Cu^{2+}+2L^{3-}=Cu_2L_2^{2-}$	16.9
CuL_2^{4-}	$Cu^{2+}+2L^{3-}=CuL_2^{4-}$	8.9

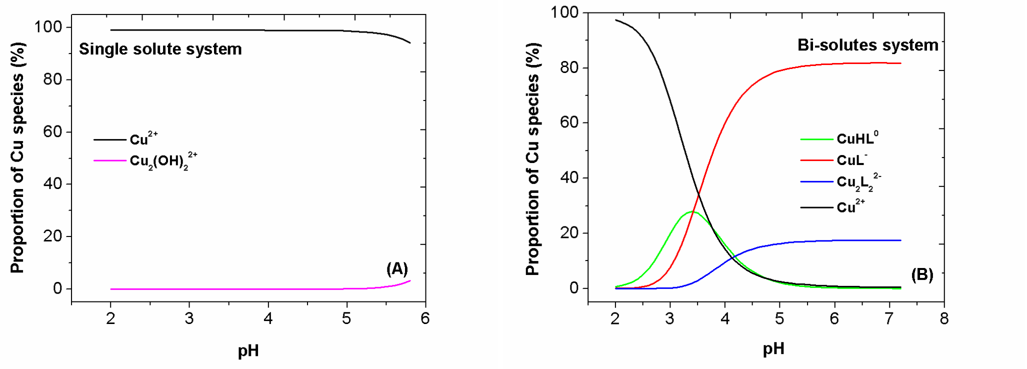

Figure S2. Distribution of Cu species in single solute system (A) and bi-solutes system (B) at different aqueous pH values. Initial concentration of Cu and CA were both 2 mmol/L.

<u>Discussion</u>: In single solute system, more than 94% of Cu was ligand-free copper ions

(Cu^{2+}) at pH below 5.8, then $Cu(OH)_2$(s) would precipitate out at higher pH value. For bi-solutes system, besides Cu^{2+}, [Cu-CA] complex species ($CuHL^0$, CuL^- and $Cu_2L_2^{2-}$) was also formed at pH between 2.0 and 7.0, holding 10~90% concentration of total Cu species. Furthermore, due to the presence of CA, Cu species was all dissolved at pH lower than 7.2 in bi-solutes system.

Table S3. Distribution of Cu species under various initial concentration ratio of CA and Cu. Initial concentration of Cu ($C_{0, Cu}$) was fixed at 2.0 mmol/L, initial concentration of CA ($C_{0, CA}$) ranged from 0 to 4.0 mmol/L; Initial pH was 4.0.

Species (%)	$C_{0, CA}$: $C_{0, Cu}$				
	0:1	0.25:1	0.5:1	1:1	2:1
Cu^{2+}	99.8	75.9	52.2	13.6	2.1
$CuHL^0$	-	4.9	9.4	16.3	18.1
CuL^-	-	18.4	35.1	60.6	67.7
$Cu_2L_2^{2-}$	-	0.9	3.2	9.2	11.8

Examination with ESI-MS

To examine the above results, bi-solutes solution of Cu and CA at equimolar initial concentration was analyzed with ESI-MS (LCQ Fleet ESI Mass Spectrometer, USA).

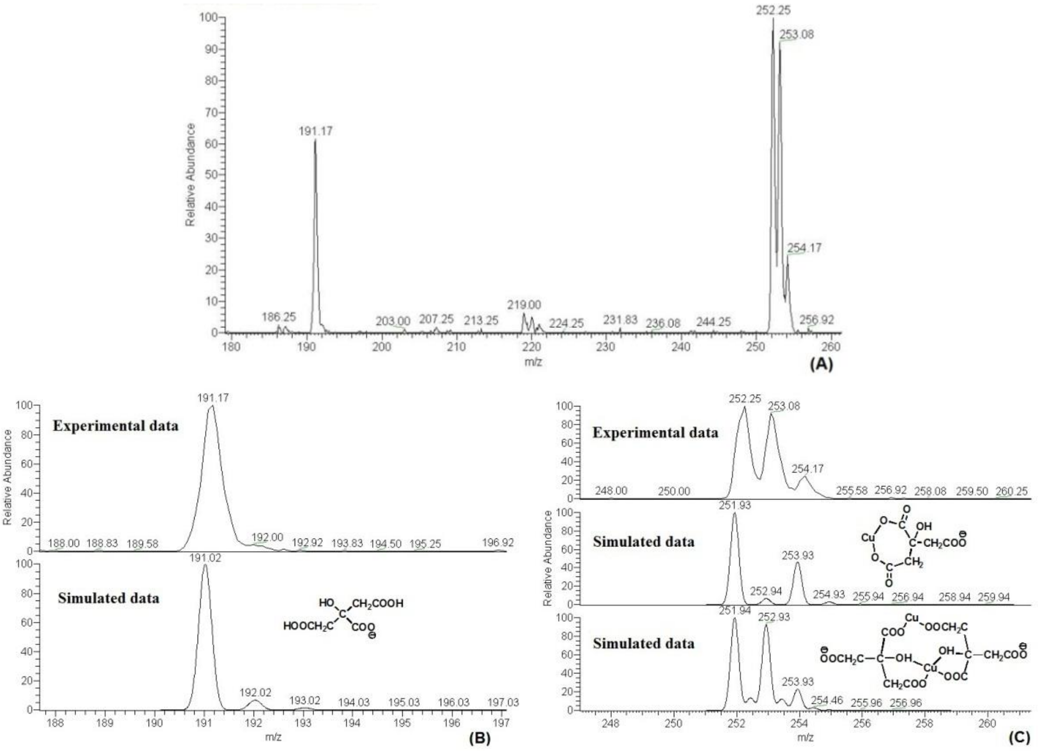

Figure S3. ESI-MS Spectra of bi-solutes solution. Initial concentrations of Cu and CA were both 2 mmol/L; Initial pH was 4.0.

__Discussion__: According to results of species calculation, the simulated [M/Z] values of H_2L^-, CuL^- and $Cu_2L_2^{2-}$ are respectively 191.0, 252.55 and 252.55. As expected, peaks at [M/Z] values around 191.1and 252-254 were markedly detected in Figure S3, which directly confirmed the existence of anionic complex species (CuL^- and $Cu_2L_2^{2-}$). In addition, $CuHL^0$ was also present since it should break up into ions such as CuL^- and H_2L^-.

Complexation structure and DFT optimization

According to Kabra et al[3], and Francis et al[4], Cu^{2+} could form either mononuclear or binuclear complex with CA molecules/ions. The former involved two carboxylic and one hydroxyl group, while the latter four carboxylic acid group and two hydroxyl groups. These structures of complexes were optimized by the DFT method for the minimum energy with single point energy calculations at the B3LYP/6-311++G(d, p)//B3LYP/6-31+G(d) (LANL2DZ for metal ions) level. The optimized structures and detail geometrical parameters were shown in Figure S4 and Table S4.

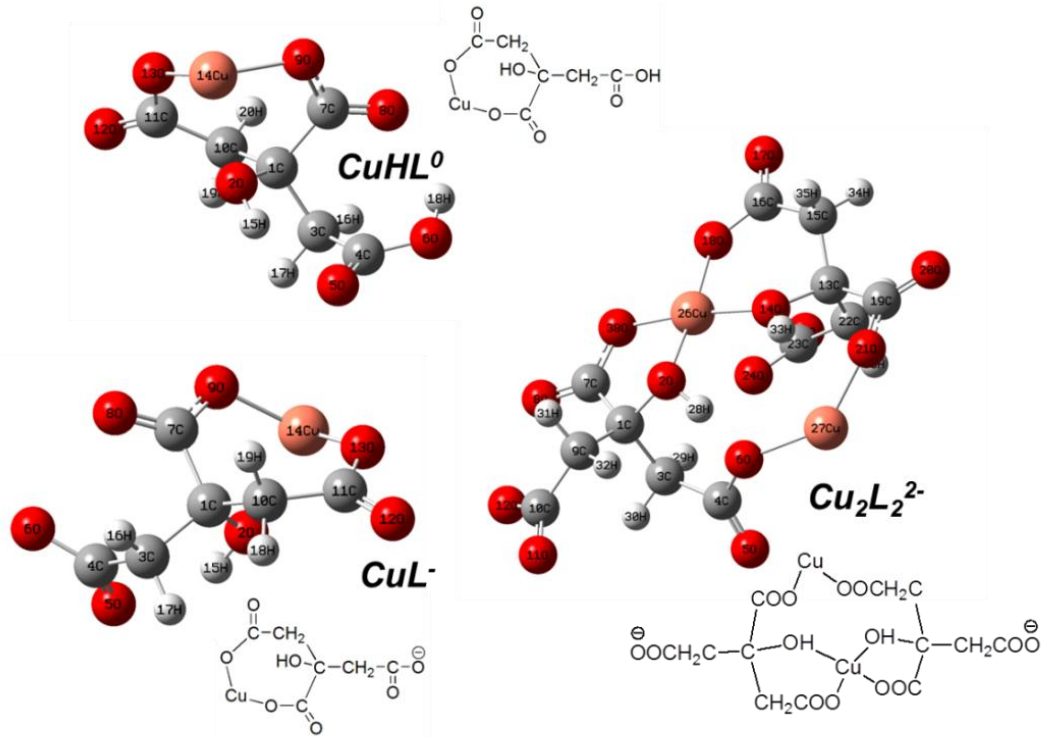

Figure S4. Optimized structures of [Cu-CA] complexes.

Table S4 Selected optimized geometrical parameters of complexes

	CuL^-		$Cu_2L_2^{2-}$	
Bond length (Å)			Cu27-O21	1.93
			Cu27-O6	1.95
	Cu14-O9	1.90	Cu26-O14	1.90
	Cu14-O13	1.85	Cu26-O2	1.88
			Cu26-O18	1.88
			Cu26-O38	1.88
Bond angle (°)			O21-Cu27-O6	138.04
			O14-Cu26-O2	92.13
	O9-Cu14-O13	109.64	O14-Cu26-O18	89.49
			O18-Cu26-O38	95.22
			O2-Cu26-O38	83.70
Dihedral angle (°)			O40-C7-C1-O2	16.89
	Cu14-O9-C7-C1	3.37	Cu26-O18-C16-C1	52.33
	Cu14-O13-C11-C10	-27.97	O18-Cu26-O14-C13	-33.15
	C11-O13-Cu14-O9	61.12	C13-C19-O21-Cu27	39.29
			C3-C4-O6-Cu27	-133.56

<u>Discussion</u>: Hydroxyl group was hard to participate in the coordination in mononuclear complex ($CuHL^0$ and CuL^-) because of long spatial distance. Then in binuclear complex one Cu ion was quadridentate-coordinated by two carboxylic groups and two hydroxyl groups, while the other Cu ion was bidentate-coordinated by only two carboxylic groups.

Part III Supplementary experiments

Resin Comparison

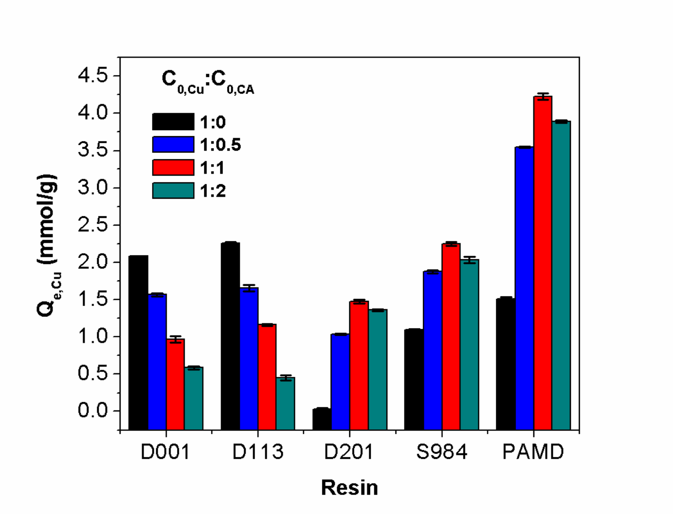

Figure S5. Effect of CA on Cu adsorption by PAMD and commercial resins. Resin dosage: 25 mg (dry weight basis), 100 mL; Initial concentration of Cu was fixed at 2.0 mmol/L, initial concentration of CA ranged from 0 to 4.0 mmol/L; Initial pH was 4.0; Under 303 K, 48h.

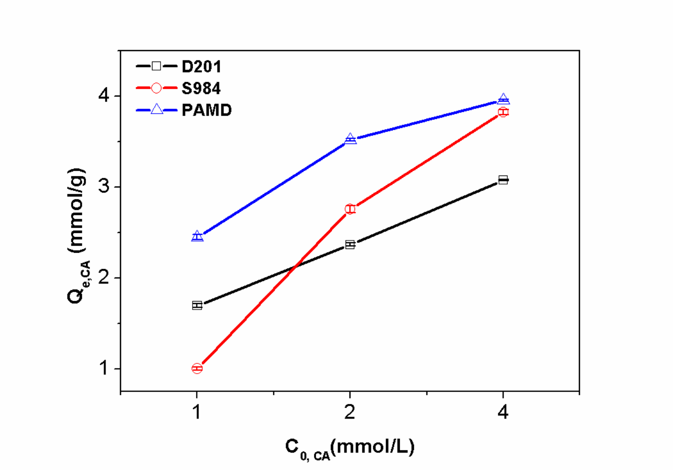

Figure S6. CA adsorption on PAMD and commercial resins in bi-solutes system. Resin dosage: 25 mg (dry weight basis), 100 mL; Initial concentration of Cu was fixed at 2.0 mmol/L, initial concentration of CA ranged from 1.0 to 4.0 mmol/L; Initial pH was 4.0; Under 303 K, 48h.

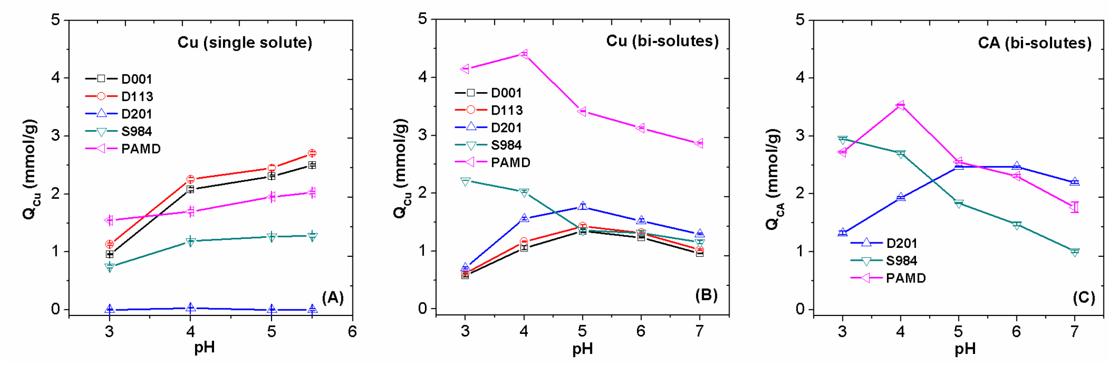

Figure S7. Cu adsorption in single solute system (A) and bi-solutes system (B), CA adsorption amount in bi-solutes system (C) as a function of aqueous pH-values. Resin dosage: 25 mg (dry weight basis), 100 mL; Initial concentrations of Cu and CA were both fixed at 2.0 mmol/L; Initial pH-values were set as 3.0-5.5 in single solute system, 3.0-7.0 in bi-solutes system; Under 303 K, 48h.

Discussion: With the increasing of initial pH-values, Cu adsorption onto D001, D113, S984 and PAMD all gradually increased in single solute systems due to more sites deprotonated. However Cu uptake in bi-solutes system generally rose first then dropped along with pH increasing. For PAMD, the adsorption of Cu and CA were both maximum at pH 4.0, which was probably attributed to the best match of distributions of solute species and amine site species at pH 4.0. With the increase of aqueous pH, anionic species increased but positive amine sites decreased. Overall, the presence of CA markedly facilitated Cu adsorption under all tested pH-values, and PAMD consistently showed the largest capacities for Cu among the five resins in all bi-solutes systems.

CA release from PAMD in preloaded system

In CA preloaded tests, a control group of CA preloaded resin was added in ultrapure water (pre-adjusted to pH 4.0) for comparison. Then after equilibrium (48h), CA concentrations in Cu solutions and water were all detected. The enhancement amount of Cu adsorption (EA, calculated as Eq. S1) and CA release ($Q_{CA, re}$, calculated as Eq. S2) as a function of CA preloaded amount was shown in Figure S8.

$$EA = Q_{Cu,pre} - Q_{Cu,cont} \cdots (Eq. S1)$$

$$Q_{CA,re} = \frac{C_{CA,re} \times V}{m} \cdots (Eq. S2)$$

Where, $Q_{Cu, pre}$ and $Q_{Cu, cont}$ are adsorption amounts in preloaded system and control single solute system (mmol/g). $C_{CA, re}$ is the concentration of CA in Cu solution or water at equilibrium (mmol/L), and m is the mass of resin (g), V is the volume of solution (L).

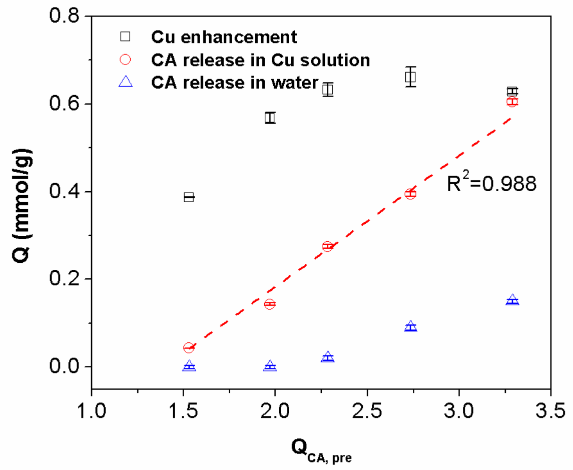

Figure S8. Enhancement amount of Cu adsorption and CA release amount as a function of CA preloaded amount.

Kinetic studies for systems with excess CA or excess Cu

(1) Excess CA ($C_{0,Cu}$=4.0 mmol/L, $C_{0,CA}$=6.0 mmol/L)

For system containing excess CA, the kinetic adsorption curve and concentration variations of each species were shown in Figure S9 and related parameters were listed at Table S5.

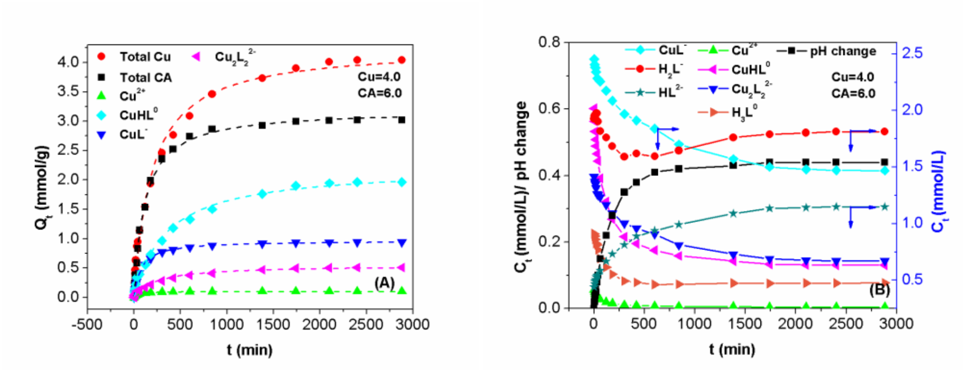

Figure S9. Kinetic curves for adsorption of total Cu, total CA and major species (A), and variations of major species concentrations and solution pH (B) in bi-solutes system containing excess CA. Adsorption data are fitted by second-order kinetic model (dash lines). Resin dosage: 250 mg (dry weight basis), 1000 mL; Initial concentrations of Cu and CA are 4.0 and 6.0 mmol/L, respectively; Initial pH was 4.0; At 303 K, 48h.

Table S5 Kinetic parameters for adsorption of Cu and CA onto PAMD in bi-solutes system with excess CA.

		Pseudo-first-order model			Pseudo-second-order model			
**Targets**	$Q_{e,\,exp}$	$Q_{e,\,fit}$	k_1	R^2	$Q_{e,\,fit}$	k_2	h	R^2
Total Cu	4.04	3.84	3.81E^{-3}	0.968	4.27	1.21E^{-3}	2.21E^{-2}	0.990
Total CA	3.02	2.92	6.51E^{-3}	0.991	3.20	2.7E^{-3}	2.76E^{-2}	0.998
Cu^{2+}	0.10	0.10	1.63E^{-2}	0.968	0.11	2.16E^{-1}	2.61E^{-3}	0.996
$CuHL^0$	0.94	0.89	1.01E^{-2}	0.978	0.97	1.33E^{-2}	1.25E^{-2}	0.998
CuL^-	1.97	1.90	2.38E^{-3}	0.978	2.22	1.30E^{-3}	6.41E^{-3}	0.990
$Cu_2L_2^{2-}$	0.51	0.49	3.14E^{-3}	0.970	0.55	7.35E^{-3}	2.22E^{-3}	0.989

Discussion: For system with excess CA, the initial concentrations of main species were in the order of CuL^- (2.45 mmol/L)> H_2L^- (1.92 mmol/L)> $CuHL^0$ (0.60 mmol/L)> HL^{2-} (0.44 mmol/L)> $Cu_2L_2^{2-}$ (0.40 mmol/L)> H_3L^0 (0.22mmol/L)> Cu^{2+} (0.06 mmol/L). Similar to the case in system at equimolar ratio, the concentrations of four Cu species and H_3L^0 dropped with adsorption time while that of HL^{2-} increased to 1.14mmol/L and H_2L^- dropped first then rose later. Moreover, the adsorption of Cu^{2+} and $CuHL^0$ were also much faster than that of CuL^- and $Cu_2L_2^{2-}$, and the magnitudes of k_2 for Cu^{2+} and $CuHL^0$ was higher than those for CuL^- and $Cu_2L_2^{2-}$, proving two interaction mechanisms. Furthermore, the adsorption amount of each species also confirmed the quantitative relationship in Eq.6.

(2) Excess Cu ($C_{0,Cu}$=4.0 mmol/L, $C_{0,CA}$=2.0 mmol/L)

For system containing excess Cu, the kinetic adsorption curve and concentration variations of each species were shown in Figure S10 and related parameters were listed at Table S6.

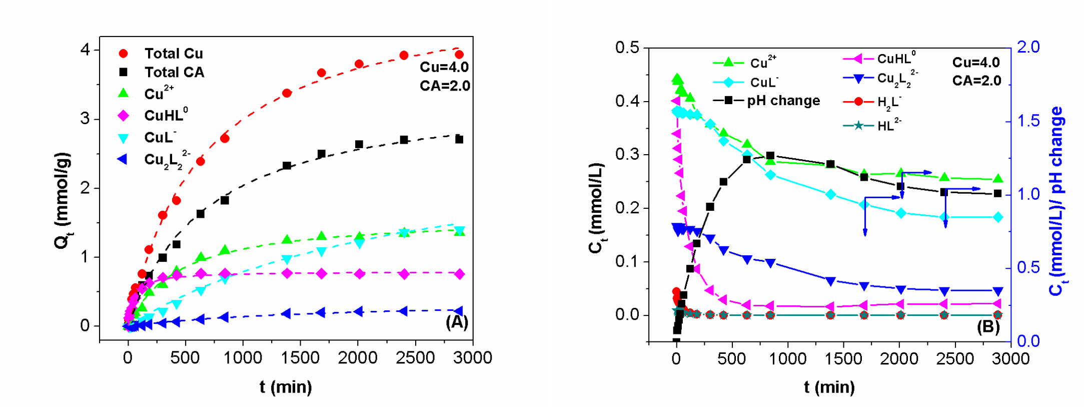

Figure S10. Kinetic curves for adsorption of total Cu, total CA and major species (A) and variations of major species concentrations and solution pH (B) in bi-solutes system containing excess Cu. Adsorption data are fitted by second-order kinetic model (dash lines). Resin dosage: 250 mg (dry weight basis), 1000 mL; Initial concentrations of Cu and CA are 4.0 and 2.0 mmol/L, respectively; Initial pH was 4.0; Under 303 K, 48h.

Table S6. Kinetic parameters for adsorption of Cu and CA onto PAMD in bi-solutes system with excess Cu.

		Pseudo-first-order model			Pseudo-second-order model			
**Targets**	$Q_{e, exp}$	$Q_{e, fit}$	k_1	R^2	$Q_{e, fit}$	k_2	h	R^2
Total Cu	3.91	3.94	$1.56E^{-3}$	0.993	4.94	$3.14E^{-4}$	$7.66E^{-3}$	0.996
Total CA	2.69	2.73	$1.49E^{-3}$	0.990	3.43	$4.25E^{-4}$	$5.00E^{-3}$	0.992
Cu^{2+}	1.33	1.33	$2.17E^{-3}$	0.995	1.40	$4.45E^{-3}$	$8.72E^{-3}$	0.995
$CuHL^0$	0.76	0.75	$1.34E^{-2}$	0.983	0.79	$2.42E^{-2}$	$1.51E^{-2}$	0.992
CuL^-	1.36	1.85	$5.24E^{-4}$	0.997	2.83	$1.38E^{-4}$	$1.11E^{-4}$	0.997
$Cu_2L_2^{2-}$	0.23	0.26	$8.25E^{-4}$	0.997	0.63	$1.59E^{-3}$	$6.31E^{-4}$	0.996

Discussion: For system with excess Cu, the initial concentration of main species was in the order of Cu^{2+} (1.79 mmol/L)> CuL^- (1.57 mmol/L)>> $CuHL^0$ (0.40 mmol/L)> $Cu_2L_2^{2-}$ (0.16 mmol/L)> H_2L^- (0.04 mmol/L)> HL^{2-} (0.01 mmol/L). Both two kinetic model fitted well. Adsorption of Cu^{2+} and $CuHL^0$ were also faster than that of two negative Cu species with higher values of adsorption rate constant (k_1 or k_2). However, there was no obvious increase of the concentration of HL^{2-} with the extension of adsorption time. The different trend was probably caused by the bridging interaction: large amount of Cu^{2+} loaded onto PAMD through coordination with neutral amine sites could create dense Cu sites for free CA species (like HL^{2-}) binding to the resin[5]. Therefore, the difference between Q_{Cu} and Q_{CA} decreased and was smaller than the sum of $Q_{Cu^{2+}}$ and Q_{CuHL^0}.

The above investigations suggested that the behavior and mechanism of Cu adsorption in various bi-solutes systems was generally consistent. Cu was adsorbed as four main species (Cu^{2+}, $CuHL^0$, CuL^- and $Cu_2L_2^{2-}$). Cu^{2+} and $CuHL^0$ coordinated with neutral amine sites, while CuL^- and $Cu_2L_2^{2-}$ interacted with protonated amine sites via electrostatic attraction. The mechanism change from single site affinity to dual-sites interaction was the reason for the great enhancement of Cu adsorption capacity onto PAMD in the presence of CA.

XPS

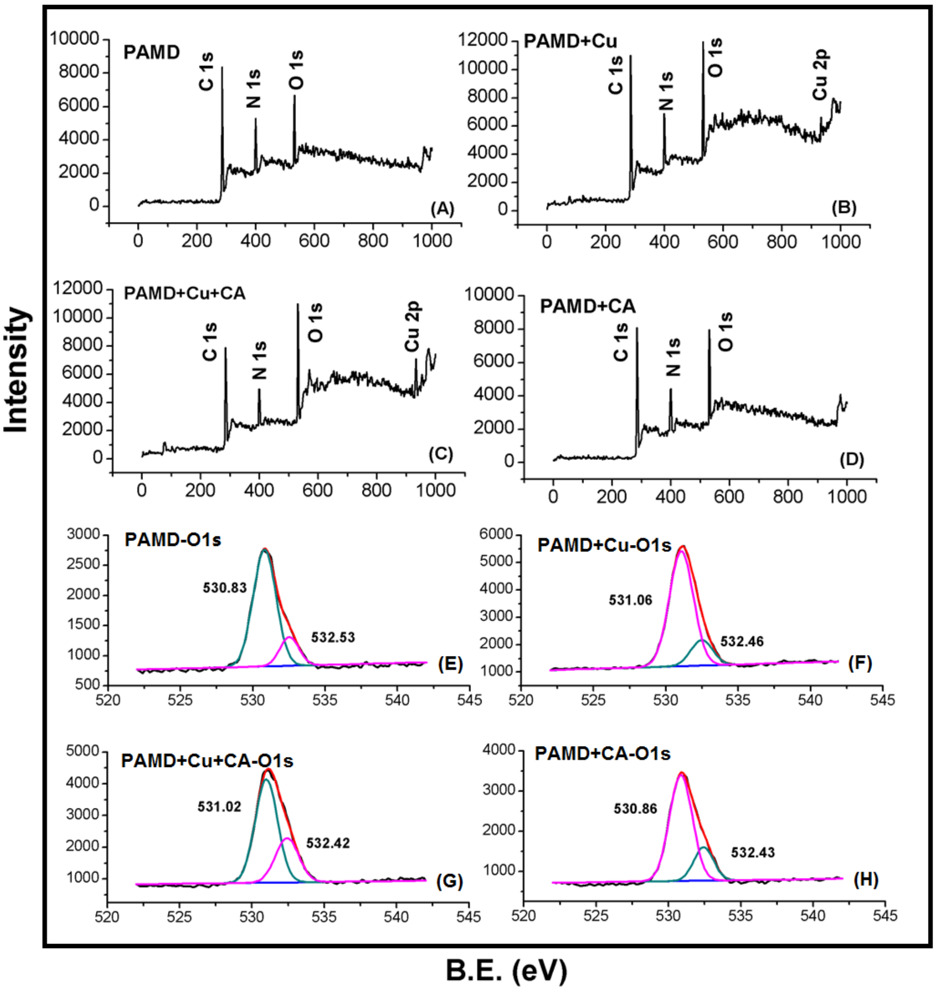

Figure S11. XPS spectra (wide scan and O1s) of PAMD (A, E), PAMD+Cu (B, F), PAMD+Binary (C, G) and PAMD+CA (D, H).

Effect of common ions

The four commercial resins and PAMD were synchronously tested in single solute systems and bi-solutes systems in the background of NaCl (20mmol/L), $Ca(NO_3)_2$ (10mmol/L), Na_2SO_4 (10 mmol/L) and K_3PO_4 (2 mmol/L). The concentrations of salt ions were set according to the common real wastewaters. All the other experiment conditions were the same with those in batch adsorption equilibrium studies. A parallel set of controls without these salt ions was also conducted. The results are presented in Figure S12.

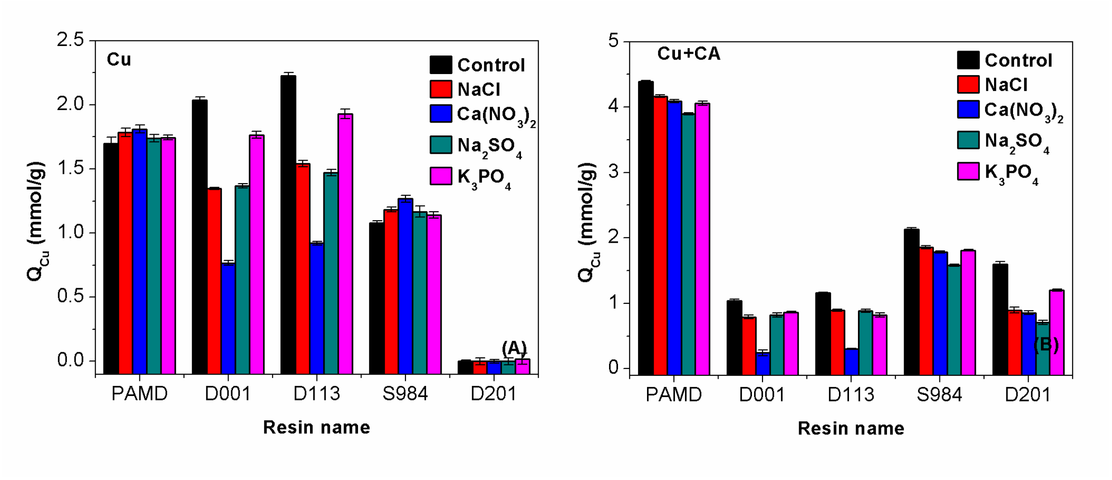

Figure S12. Effect of common ions on Cu adsorption by different resins in single solute systems and bi-solutes systems (25 mg dry rein, 100 mL; $C_{0,\,Cu}$ was all fixed at 2.0 mmol/L, $C_{0,CA}$ was 2.0 mmol/L in bi-solutes system; the concentrations of NaCl, $Ca(NO_3)_2$, Na_2SO_4 and K_3PO_4 were 20, 10, 10 and 2 mmol/L, respectively. The controls represented the cases without these salts. Initial pH was all 4.0; Under 303 K, 48h).

Fixed-bed dynamic adsorption and resin regeneration

Text S1

The saturated adsorption capacity (Q_S, mmol/g) in fixed-bed and the recovery ratio (R, %) were calculated as Eq. S3 and Eq. S4[6].

$$\mathrm{Q_S} = \int_0^{V_S}(C_0 - C_t)\frac{dV}{m} = \sum_{i=1}^{n}\frac{(C_0 - C_i)\times V_i}{m} \cdots Eq.S3$$

$$\mathrm{R} = \frac{\int_0^{V_R} C_t\frac{dV}{m}}{Q_s} \times 100\% = \frac{\sum_{i=1}^{n}\frac{C_i\times V_i}{m}}{Q_s} \times 100\% \cdots Eq.S4$$

Where, m (g) is the total amount of dry resin beads in fix-bed. C_0 and C_t (mmol/L) are the initial and effluent concentrations of pollutants (Cu or CA). V_s (L) is the volume of solution required to reach the saturation point (C_t/C_0=0.95). i is the serial number of collection tube, and C_i (mmol/L) is the average concentration in No. i tube. V_i (L) is volume of solution in the corresponding collection tube. V_R (L) is the total volume of regeneration solution. In recovery (desorption) stage, all the collected solution in all tubes was mixed together and the average concentrations of Cu and CA were 4051 mg/L and 10743 mg/L, respectively. The adsorption breakthrough and recovery curves of Cu and CA by PAMD are shown in Figure S13. The regenerated resin bed were rinsed with ultrapure water until the effluent was at neutral pH and then reused for the next round adsorption. Saturated adsorption capacities and recovery ratio of the two solutes by PAMD in the five cycles are shown in Figure S14.

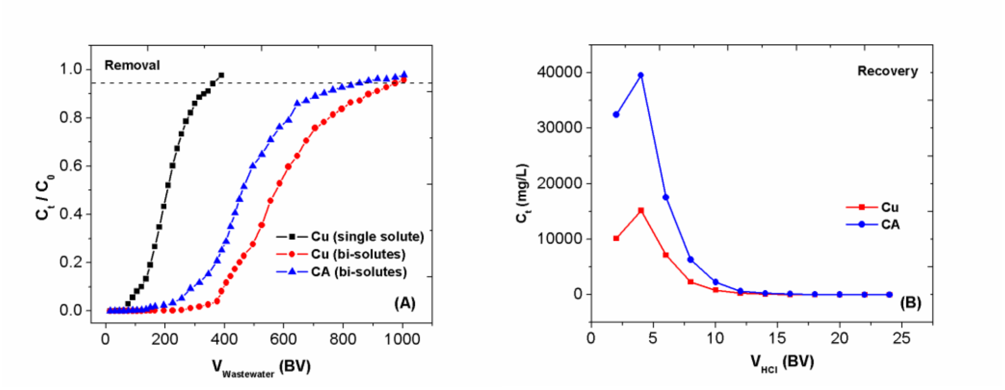

Figure S13. Dynamic adsorption of Cu and CA onto PAMD in single solute and bi-solutes systems (A) and recovery of Cu and CA from exhausted PAMD with 2 M HCl (B). 500 mg dry resin; Initial concentration of Cu and CA were both 2.0 mmol/L; Initial pH was 4.0; For adsorption, flow rate was 5 BV/h, at 303K; For recovery, flow rate was 1 BV/h, at room temperature.

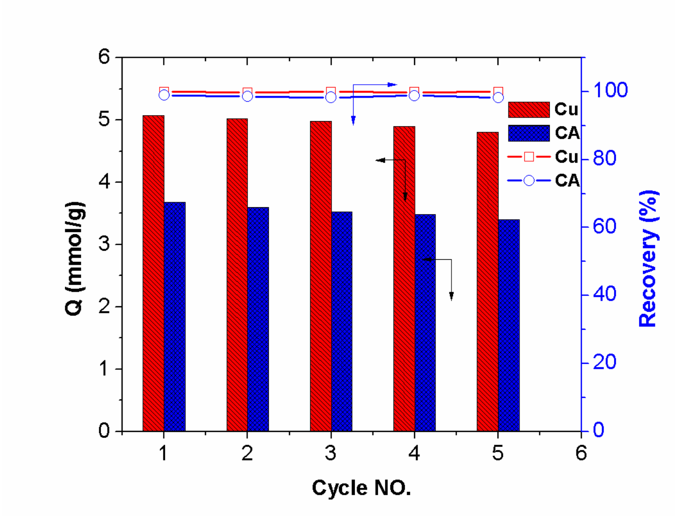

Figure S14. Adsorption−desorption cycles of PAMD.

The application of PAMD in other similar systems

To further prove the universality of large enhancement effect in our work, PAMD was also tested in other similar systems involving HMIs (Cu/ Ni/ Zn) and OAs (tartaric acid (TA)/ citric acid (CA)/ oxalic acid (XA)/ ethylenediamine tetraacetic acid (EDTA)).

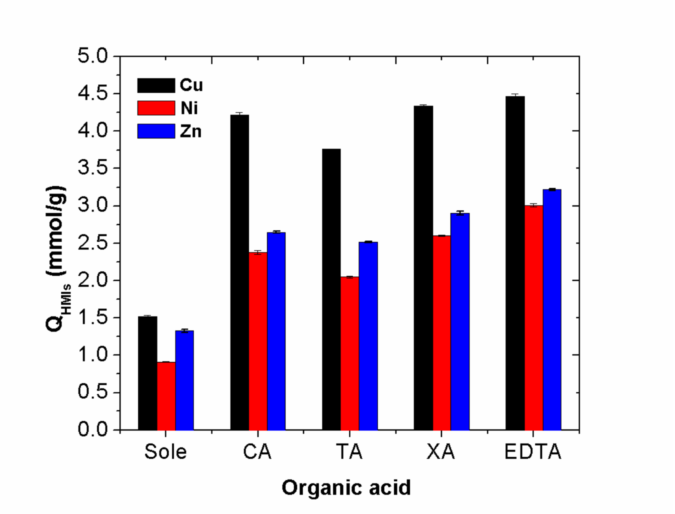

Figure S15. Adsorption of HMIs onto PAMD in presence of different OAs. Dosage: 25 mg dry resin, 100 mL; Initial concentration of each solute was all set as 2.0 mmol/L; Initial pH was 4.0; Under 303 K, 48h.

<u>Discussion</u>: As shown in Figure S15, PAMD consistently showed remarkable enhancement in HMIs adsorption from systems containing OAs. The enhancement rate (E_r) ranged from 99.6%~230.1%, which was dependent on both complexation ability of solutes and interaction mechanisms in resin-phase. For instance, the enhancement in systems containing EDTA was probably also driven by dual-sites interaction. In contrast, for TA/ XA systems, other mechanisms should be more important for the enhancement because most of aqueous complex species in their bi-solutes system were neutrally charged.

References

1 Craven, A. M., Aiken, G. R. & Ryan, J. N. Copper(II) Binding by Dissolved Organic Matter: Importance of the Copper-to-Dissolved Organic Matter Ratio and Implications for the Biotic Ligand Model. *Environ. Sci. Technol.* **46**, 9948-9955 (2012).

2 Park, S.-M., Yoo, J.-C., Ji, S.-W., Yang, J.-S. & Baek, K. Selective recovery of Cu, Zn, and Ni from acid mine drainage. *Environ. Geochem. Health* **35**, 735-743 (2013).

3 Kabra, K., Chaudhary, R. & Sawhney, R. L. Solar photocatalytic removal of Cu(II), Ni(II), Zn(II) and Pb(II): Speciation modeling of metal–citric acid complexes. *J. Hazard. Mater.* **155**, 424-432 (2008).

4 Francis, A. J., Dodge, C. J. & Gillow, J. B. Biodegradation of metal citrate complexes and implications for toxic-metal mobility. *Nature* **356**, 140-142 (1992).

5 Henry, W. D., Zhao, D., SenGupta, A. K. & Lange, C. Preparation and characterization of a new class of polymeric ligand exchangers for selective removal of trace contaminants from water. *React. Funct.Polym.* **60**, 109-120 (2004).

6 Meng, M. *et al.* Highly efficient adsorption of salicylic acid from aqueous solution by wollastonite-based imprinted adsorbent: A fixed-bed column study. *Chem. Eng. J.* **225**, 331-339 (2013).
